# Research Progress on the Anticancer Effect of Ginsenoside Rh1

**DOI:** 10.3390/cimb48020219

**Published:** 2026-02-17

**Authors:** Yiqiong Zhang, Qinghua Yao

**Affiliations:** 1 The Second School of Clinical Medicine, Zhejiang Chinese Medical University, Hangzhou 310053, China; zhyq93956149@126.com; 2 Department of Oncology, The Second Affiliated Hospital of Zhejiang Chinese Medical University, Hangzhou 310005, China

**Keywords:** ginseng, ginsenoside Rh1, cancer, anti-cancer effect, biological mechanism

## Abstract

Cancer is one of the major lethal diseases in the world, and Western medicine treatments are often affected by side effects and drug resistance. Ginseng is a commonly used Chinese medicine in clinical practice, and Ginsenoside Rh1, an important active ingredient in ginseng, has received widespread attention in recent years for its remarkable anticancer potential. In this paper, we systematically described the inhibitory effects of Ginsenoside Rh1 and its molecular mechanism in hepatocellular carcinoma, gastric carcinoma, colon carcinoma, breast carcinoma, ovarian carcinoma, cervical carcinoma, lung carcinoma and glioma. Studies have shown that Rh1 can inhibit cancer cell proliferation, migration, and invasion, and induce apoptosis by regulating multiple signaling pathways. In addition, Rh1 can inhibit MMPs expression and regulate angiogenesis and the immune microenvironment to exert synergistic anticancer effects. Although the efficacy of Rh1 has been confirmed in vitro and animal studies, the clinical translation of Rh1 requires further exploration of its in vivo pharmacokinetics, long-term safety, and precise targets. In this paper, we systematically summarize the multiple anticancer mechanisms of Rh1 and look forward to the prospect of combining Rh1 with existing therapies to provide a theoretical basis for the development of novel anticancer drugs.

## 1. Introduction

Ginseng is renowned as the “King of Herbs”, with effects such as reinforcing vital energy, restoring the pulse, preventing collapse, strengthening the spleen and lungs, generating body fluids, nourishing the blood, calming the mind, and enhancing intelligence. It is a commonly used traditional Chinese medicine in clinical practice. Modern pharmacological studies have shown that ginseng can promote the regeneration of blood cells and tissues, regulate the endocrine system, immune response, body metabolism, the central nervous system, and possess anti-inflammatory effects, which also plays a role in inhibiting and fighting cancer in various tumors [1]. Ginsenosides are among the main active ingredients responsible for ginseng’s anti-tumor properties, including multiple subtypes such as Ginsenoside Rb1, Rb2, Rc, Rd, Re, Rg1, Rg3, Rh1, Rh2, Rh3, Rg5 and Compound K [2].

Despite significant advances in modern oncology, current mainstream cancer treatments—including chemotherapy, radiotherapy, and targeted therapy [3]—still face considerable challenges. These limitations primarily include severe systemic side effects, the emergence of drug resistance, and high treatment costs, all of which adversely affect patient quality of life and survival outcomes [4]. However, natural products, with their advantages of multi-targeting, low toxicity, and wide availability, have become an important reservoir for the discovery of novel anti-cancer drugs [5]. As key active components of ginseng, ginsenosides exemplify the value of natural products, and their anti-tumor mechanisms are a focal point of research. In recent years, after extensive research on their pharmacological effects and clinical applications both domestically and internationally, it has been found that Ginsenosides exert multiple mechanisms of action on tumor cells, including inhibition of proliferation, migration, and invasion, inhibition of angiogenesis, induction of apoptosis, enhancement of immune regulation, and regulation of cell autophagy. They can also be used in combination with chemotherapeutic agents and targeted drugs to increase the sensitivity of cancer cells to anti-cancer drugs, reduce adverse reactions, and lower drug resistance [6]. The anti-cancer effects of rare subtypes of ginseng saponins are gradually being discovered.

**Figure 1:**
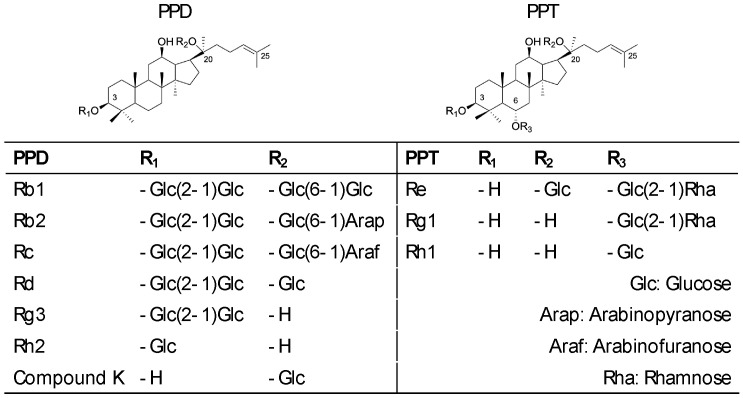
Stereochemical structures of PPD- and PPT-type ginsenosides.

There are many types of ginsenosides, which can be mainly divided into dammarane type and oleanolic type according to their glycoside structure [7]. Among them, dammarane type can be divided into protopanaxadiol (PPD) and protopanaxatriol (PPT), the former includes but is not limited to Rb, Rc, Rd, Rg3 and Rh2, and the latter includes Re, Rg1, Rh1, etc. (Figure 1) Oleanolic types include Rh3 and so on [8,9]. The antitumor activity of ginsenosides is closely associated with their structural features. In terms of aglycone type, PPD-type saponins generally exhibit stronger antitumor effects compared to PPT-type saponins. Regarding glycosylation, the number of glycan units attached inversely correlates with antitumor activity—increased glycosylation typically leads to reduced potency. Structural modifications at specific positions also significantly influence bioactivity: the introduction of hydroxyl or methoxy groups at C25 tends to enhance the cytotoxic activity of the compound, whereas dehydration at C20 increases its pharmacological efficacy. Furthermore, the stereochemistry at C20 plays a critical role, with the 20(S)-epimer generally demonstrating more potent antitumor activity than its 20(R)-counterpart. Additionally, both the position of the glycan moiety and structural modifications on the glycan itself can further modulate the biological activity of ginsenosides [10]. For example, in PPD, Rh2 and Compound K all had inhibitory effects on multiple cancer types. In multiple experiments, it was confirmed that Rh2 could up-regulate the expression of miR-200b-5p, miR-224-3p and miR-146a-5p, and down-regulate the expression of miR-26b-3p and miR-29a-5p, thus determining that Rh2 Rh2 exerts inhibitory effects on proliferation and promotes apoptosis in HepG2 liver cancer cells; in addition to this, Rh2 plays an important role in regulating β-catenin, EGFR and other pathways, and can induce ROS [11,12,13,14,15,16,17,18,19,20,21]. Compound K can inhibit STAT3 and PI3K/Akt pathways and induce autophagy [22,23,24,25,26,27,28]. While Rd and Rg5 mainly play a major role in breast, stomach and lung cancer. Rd can inhibit the Smad2 and Akt pathways, and has a special effect on enhancing chemotherapy sensitivity [29,30,31]. Rg5 can cause autophagy and G2/M blockade [32,33,34]. In PPT, Rg1 mainly inhibits liver cancer through hepatoprotective and sensitizing DNA damage drugs [35,36] (Table 1).

**Figure 2:**
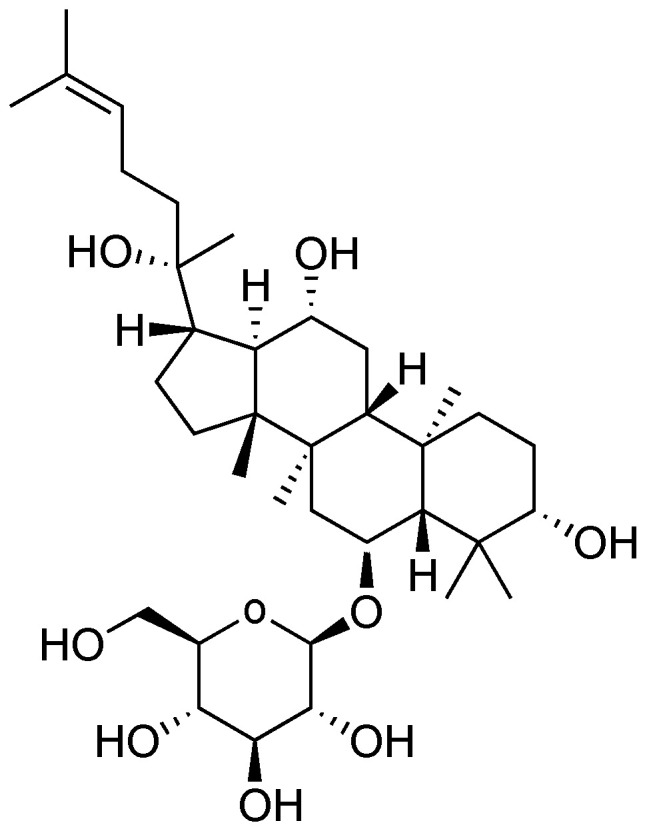
The structure of Ginsenoside Rh1.

It is noteworthy that Ginsenoside Rh1 (Figure 2), a PPT-type dammarane ginsenoside, exhibits a broad spectrum of pharmacological activities, including notable anti-inflammatory, antioxidant, anti-tumor, neuroprotective, and immunomodulatory effects [62,63]. Compared with its precursor Ginsenoside Rg1, Ginsenoside Rh1 exhibits demonstrates stronger overall and in vitro anti-tumor efficacy, highlighting its superior potential as an anti-cancer agent. Its mechanisms of action are distinct: for instance, it can simultaneously promote angiogenesis and inhibit vascular leakage by targeting and regulating NR4A1, thereby exerting preventive and therapeutic effects on various diseases involving the central nervous, cardiovascular, cerebrovascular, hematological systems, and tumors [64]. Meanwhile, compared to Rg1, Ginsenoside Rh1 exhibits a stronger inhibitory effect on colonic myeloperoxidase activity and can suppress inflammatory responses by inhibiting Th17 cell differentiation and inducing Treg cell differentiation, highlighting the unique anti-inflammatory effects of Rh1 [65]. Moreover, in combination with chemotherapy, Yang et al. [66] found that Rh1 alleviates cisplatin-induced nephrotoxicity by inhibiting the JNK/p53 pathway, enhancing the activity of human renal tubular epithelial cells, suppressing apoptosis, and reducing ROS production, providing a basis for Rh1 as an adjuvant to mitigate chemotherapy-induced toxicity.

However, existing reviews have largely focused on the ginsenoside family as a whole or on more extensively studied monomers such as Rg3 and Rh2. A systematic summary of the anti-cancer effects of Ginsenoside Rh1, particularly its distinctive mechanisms and advantages compared to other ginsenosides, remains insufficient. Given the limitations of current cancer therapies and the unique value of natural products, this review aims to comprehensively summarize recent research progress on the anti-cancer effects of Ginsenoside Rh1 based on relevant domestic and international literature, with emphasis on its characteristic mechanisms and differential effects compared to other ginsenosides, in order to provide a theoretical basis and valuable reference for the development of novel, efficient, and low-toxicity anti-cancer drugs derived from traditional Chinese medicine.

## 2. Ginsenoside Rh1 and Digestive System Tumors

### 2.1. Ginsenoside Rh1 and Hepatocellular Carcinoma

Primary hepatocellular carcinoma ranks sixth in incidence and third in mortality among malignant tumors [67]. At present, traditional Chinese medicine can be combined with Hepatic Arterial Chemoembolization, radiotherapy and chemotherapy, and Molecular Targeted Therapy, playing a positive role in reducing adverse reactions and modulating immunity.

Yoon et al. [68] conducted radioactive labeling of oligomers at the AP-1 binding site of MMP-1 in HepG2 human liver cancer cells, followed by expression analysis and electrophoretic Migration rate variation analysis. This experiment demonstrated that Rh1 significantly inhibited the migration and invasion ability of HepG2 cells at ≤100 μM non-toxic concentration, showing concentration and time dependence. They found that Ginsenoside Rh1 could inhibit the expression of Matrix Metalloproteinase-1 by attenuating the induction effect of PMA and affect the promoter activity of Matrix Metalloproteinase-1 by inhibiting the MAPK pathway, thereby suppressing the growth, invasion, and migration of human liver cancer cells related to Matrix Metalloproteinase-1 expression. This study demonstrates that Ginsenoside Rh1 has the potential to be developed as a novel chemotherapeutic agent for the treatment of malignant tumors, including early hepatocellular carcinoma associated with the expression of Matrix Metalloproteinase-1. Wang et al. [69] demonstrated that Ginsenoside Rh1 targets and suppresses the glucocorticoid receptor, thereby upregulating MHC-I expression. This action promotes the maturation of dendritic cells and activates CD8^+^ T cells, leading to an improved immune microenvironment and a potentiated anti-HCC effect of lenvatinib.

### 2.2. Ginsenoside Rh1 and Gastric Cancer

Multiple studies have confirmed the association between the development and progression of gastric cancer and the dysregulation of various signaling pathways. Existing research indicates that the TGF-β/Smad pathway can induce Epithelial–Mesenchymal Transition in stomach cancer cells and plays a promotive role in stomach cancer [70]. Yang et al. [71] used Oxaliplatin as a positive control and treated AGS Human Gastric Adenocarcinoma Cells with different concentrations of Ginsenoside Rh1 for 48 h. Various assays were conducted to evaluate proliferation, migration, invasion, and apoptosis. Furthermore, AGS cells were treated with a combination of Ginsenoside Rh1 and a TGF-β/Smad pathway activator to assess the impact of the TGF-β/Smad pathway on the biological behavior of AGS Human Gastric Adenocarcinoma Cells. Experiments have shown that Ginsenoside Rh1 inhibits the proliferation, migration, invasion, and apoptosis of AGS Human Gastric Adenocarcinoma Cells by suppressing the TGF-β/Smad pathway. In in vitro experiments, the effect of Ginsenoside Rh1 at a concentration of 50 μM was comparable to that of Oxaliplatin.

### 2.3. Ginsenoside Rh1 and Colorectal Cancer

Colorectal Cancer is the third most common tumor worldwide and ranks second in mortality [67]. Active ingredients in traditional Chinese medicine, represented by Flavonoids, Phenolics, and Alkaloids, can play roles in the prevention and treatment of colorectal cancer, such as Inhibition of Proliferation, Induction of Apoptosis, regulation of autophagy, inhibition of Invasion and metastasis, and suppression of angiogenesis. Compared with Western medical treatments such as surgery and chemoradiotherapy, which may cause disruption of the Intestinal Microbiota in colorectal cancer patients, traditional Chinese medicine can assist in the treatment of intestinal cancer patients by modulating the Intestinal Microbiota.

Wei et al. [72] similarly cultured SW480 Cells in the same concentration of Ginsenoside Rh1 as used in the aforementioned study, and assessed the effects of various concentrations of Ginsenoside Rh1 on the invasion and migration abilities of SW480 Cells, as well as the differences in Matrix Metalloproteinase-9 protein expression. They found that Ginsenoside Rh1 could inhibit the Migration and Invasion of SW480 Cells by suppressing the expression of Matrix Metalloproteinase-9.

**Figure 3:**
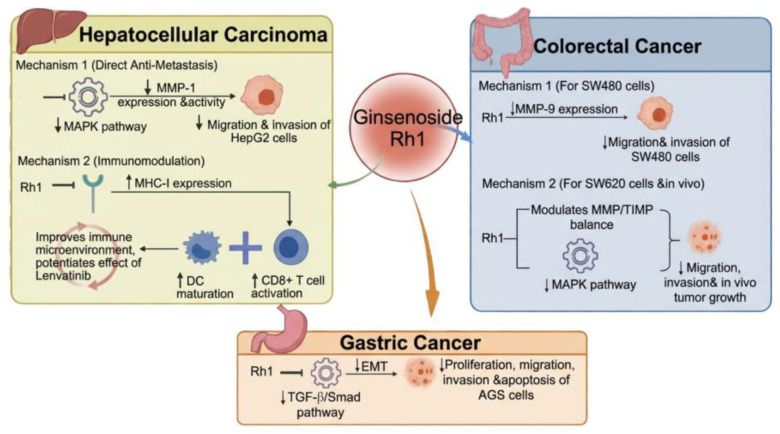
Ginsenoside Rh1 against digestive system tumors. Created in BioRender. Z, Y. (2026) https://BioRender.com/4qv41os (accessed on 5 January 2026).

Lyu et al. [73] subsequently selected SW620 cells for in vitro experiments and established a SW620 cell xenograft tumor model in nude mice to conduct in vivo studies. Their results demonstrated that Rh1 effectively inhibits cancer cell migration, invasion, and in vivo tumor growth in colorectal cancer by modulating the MMP/TIMP balance and suppressing the MAPK signaling pathway (Figure 3).

## 3. Ginsenoside Rh1 and Gynecological Tumors

### 3.1. Ginsenoside Rh1 and Breast Cancer

The efficacy of Ginsenoside Rh1 against HER2-positive breast cancer SKBR3 cells has been confirmed by multiple cellular experiments. Li et al. [74] performed enrichment analysis on the differentially expressed genes identified in the preliminary screening and found that, besides the pathways mentioned earlier, these genes were also significantly enriched in pathways such as basal adhesion, MAPK signaling pathway, and Ubiquitin-Proteasome pathway. This suggests that Ginsenoside Rh1 might inhibit SKBR3 cell activity through these pathways. Additionally, Li et al. [75] used the CCK-8 cell proliferation assay to treat SKBR3 cells with varying concentrations of Ginsenoside Rh1. The results showed that Ginsenoside Rh1 effectively inhibited cell proliferation and promoted apoptosis. Furthermore, the mRNA and protein levels of Mucin in the cells were reduced, indicating that the mechanism of action could involve inhibiting Mucin expression.

For ER+ breast cancer, Diem et al. [76] treated MCF-7, HCC1428, and BT474 cells with varying concentrations of Ginsenoside Rh1 and observed that Rh1 suppressed their proliferation. Among these cell lines, MCF-7 cells demonstrated the highest sensitivity (IC_50_ ≈ 90.28 μM). This study demonstrated that Rh1 exerts its effects through the ROS-mediated inhibition of the PI3K/Akt signaling pathway, thereby inducing apoptosis, autophagy, and cell cycle arrest. Furthermore, although Ginsenoside Rh1 exhibits weak intrinsic estrogenic activity, it displays a competitive anti-proliferative effect in the presence of estrogen. Given its low toxicity in normal cells, Rh1 represents a promising candidate for the treatment of breast cancer.

Jin et al. [77] treated Triple-negative Breast Cancer cells MDA-MB-231 with different concentrations of Ginsenoside Rh1 and used various experimental techniques to observe its effects on migration, invasion, and apoptosis, as well as to explore the underlying molecular mechanisms. The study found that Ginsenoside Rh1 could generate mitochondrial reactive oxygen species, thereby further inhibiting the activation of STAT3 and NF-κB signaling pathways, leading to downregulation of Matrix Metalloproteinases and Vascular Endothelial Growth Factor. This significantly inhibited migration and invasion of MDA-MB-231 cells and induced apoptosis. These results reveal the potential application value of Ginsenoside Rh1 in treating Triple-negative Breast Cancer. Zheng et al. [78] emphasized that breast cancer metastasis is a leading cause of mortality, with tumor cell extravasation being a critical step in the metastatic cascade. In their study, MDA-MB-231 cells were treated with varying concentrations of Ginsenoside Rh1 for in vitro experiments, while mouse and zebrafish models were established for in vivo validation. The results demonstrated that Ginsenoside Rh1, functioning as a novel natural inhibitor of CK2α, disrupts the interaction between tumor cells and endothelial cells by modulating the HHEX/CCL20 signaling axis. Consequently, this mechanism significantly inhibits the extravasation and metastasis of breast cancer cells.

### 3.2. Ginsenoside Rh1 and Ovarian Cancer

The therapeutic effects of ginsenosides on ovarian cancer are associated with multiple signaling pathways, but current studies focusing on Ginsenoside Rh1 remain limited. Sun and Xiao [79] used the MTT assay to evaluate the effects of different concentrations of Ginsenoside Rh1 on the ovarian cancer cell line SKOV3 cells. The experimental results showed that Ginsenoside Rh1 significantly inhibited the proliferation of SKOV3 cells, and to a certain extent downregulated the expression of the PIWI gene, although no dose- or time-dependent effects were observed.

### 3.3. Ginsenoside Rh1 and Cervical Carcinoma

**Figure 4:**
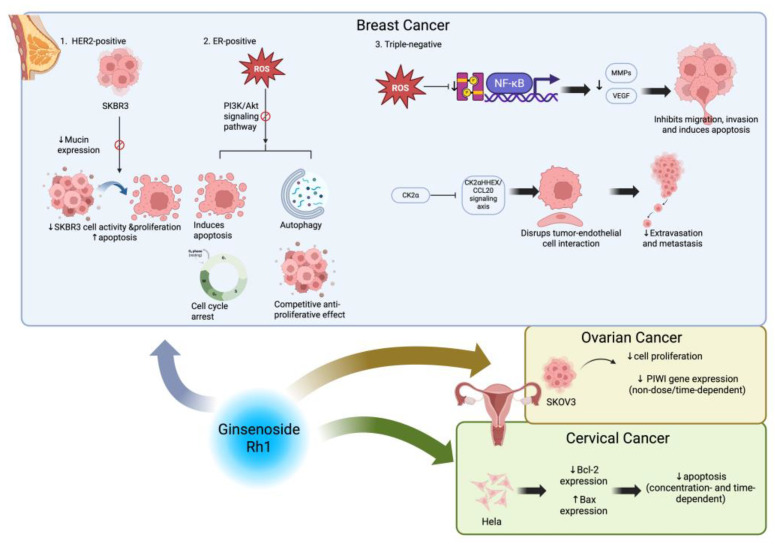
Ginsenoside Rh1 against gynecological tumors. Created in BioRender. Z, Y. (2026) https://BioRender.com/4qv41os (accessed on 5 January 2026).

The inhibitory effect of Ginsenoside Rh1 on the proliferation of cervical carcinoma cells and its ability to induce apoptosis were confirmed by Sun [80]. HeLa cervical cancer cell line was treated with different concentrations of Ginsenoside Rh1 and apoptosis was detected. The experiment found that Ginsenoside Rh1 could significantly induce apoptosis in HeLa cells by downregulating the expression of Bcl-2 Gene and promoting the expression of Bax gene, showing a concentration- and time-dependent manner (Figure 4).

## 4. Ginsenoside Rh1 and Lung Cancer

Lung cancer is the most common malignant tumor worldwide, ranking first in both incidence and mortality [67], which is closely related to smoking [81]. In order to discover more anticancer approaches from the perspective of traditional Chinese medicine, the effects of ginsenosides in Ginseng have been extensively studied.

**Figure 5:**
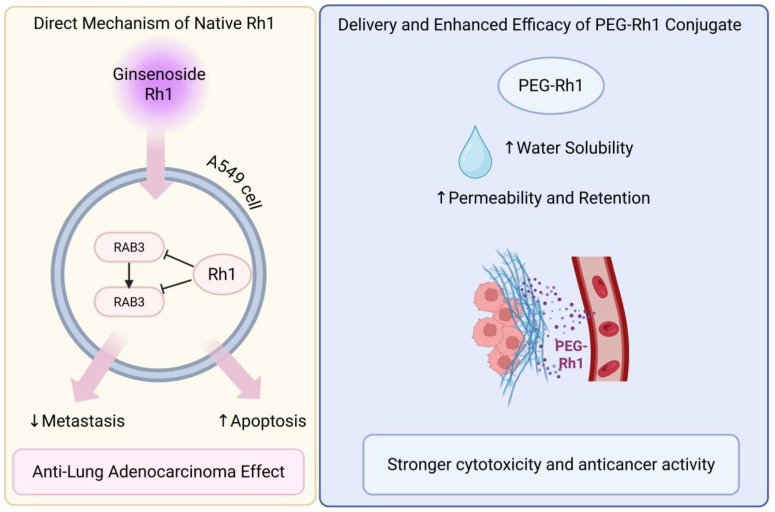
Ginsenoside Rh1 against lung cancer. Created in BioRender. Z, Y. (2026) https://BioRender.com/4qv41os (accessed on 5 January 2026).

Nahar [82] treated A549 cells with different concentrations of Ginsenoside Rh1, through computer simulation and in vitro experiments, finding that Ginsenoside Rh1 can inhibit the RhoA/ ROCK1 signaling pathway, thereby preventing and treating lung adenocarcinoma by regulating metastasis and apoptosis. Mathiyalagan [83] synthesized a conjugate of PEG and Ginsenoside Rh1 to enhance its water solubility and passive targeted delivery. In vitro cytotoxicity assays demonstrated that PEG-Ginsenoside Rh1 exhibited stronger anticancer activity in human Non-small Cell Lung Cancer cell line A549 Cells (Figure 5).

## 5. Ginsenoside Rh1 and Glioma

**Figure 6:**
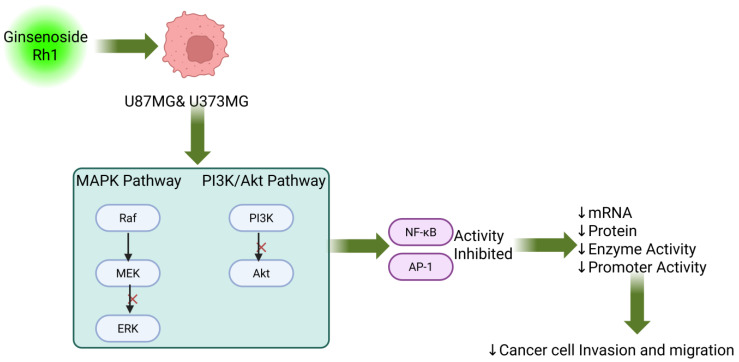
Ginsenoside Rh1 against glioma. Created in BioRender. Z, Y. (2026) https://BioRender.com/4qv41os (accessed on 5 January 2026).

Glioma is an infiltrative tumor with poor prognosis and limited treatment options, and MMPs are considered to be closely associated with the Migration and Invasion of glioma [84]. Jung et al. [85] found that Ginsenoside Rh1 can inhibit the mRNA expression, protein expression, enzyme activity, and promoter activity of PMA-induced MMP-1, -3, and -9 in human astrocytoma U87MG and U373MG cells by suppressing the MAPK and PI3K/ Akt signaling pathways, as well as downstream transcription factors such as NF-κB and AP-1, thereby effectively inhibiting their Invasion and Migration (Figure 6).

## 6. Summary and Outlook

Currently, cancer remains the leading cause of death worldwide. As a core bioactive compound derived from ginseng, ginsenoside Rh1 has garnered considerable research interest in recent years for its potential in cancer therapy [86]. Studies indicate that ginsenoside Rh1 exhibits distinct effects in suppressing cancer cell migration and invasion. Furthermore, it exerts multiple anticancer activities, including inhibiting tumor cell proliferation, inducing apoptosis, attenuating angiogenesis, and enhancing immune responses. These actions are mediated through the modulation of various signaling pathways [87,88,89,90], highlighting the promising therapeutic potential of ginsenoside Rh1 in oncology.

However, despite promising progress in preclinical research, the translation of ginsenoside Rh1 into a clinically available anticancer agent remains challenging. Current research on Rh1 is predominantly confined to the preclinical stage, relying on in vitro cell studies and animal models. Although certain formulated products, such as ginseng H drop pills [91] and various health supplements, contain ginsenosides, Rh1 is not the principal component in these preparations. The further development of ginsenoside Rh1 is constrained by several factors, including a lack of clinical trial data, high costs associated with purification and standardized production, as well as insufficient pharmacokinetic and pharmacodynamic studies. Given that the isolation of ginsenoside Rh1 is notably challenging, synthetic approaches may represent a more efficient route to obtain Rh1 and its derivatives. Future research should prioritize the elucidation of more precise molecular targets and mechanisms of action, with particular emphasis on effects that distinguish Rh1 from other ginsenosides—such as chemical sensitization and toxicity mitigation. Concurrently, comprehensive studies on its long-term safety and potential side effects are essential to ensure its reliability in future clinical applications.

In summary, Ginsenoside Rh1 presents a promising candidate for anti-cancer drug development owing to its multi-target mechanisms of action. Its distinct pharmacological profile, particularly regarding metastasis suppression and chemosensitization, sets it apart from other ginsenosides. With continued and in-depth investigation, further exploration of the full therapeutic potential of Ginsenoside Rh1 is anticipated, which may facilitate its translation from preclinical research to clinical application, ultimately providing novel therapeutic options and potential improvements in outcomes for cancer patients.

## Figures and Tables

**Table 1:** Some of the ginsenoside analogues and their main activities.

Types of Ginsenosides			Main Activities
dammarane type	PPD	Rb1	Diabetes [37], nervous [38], cardiovascular [39], and endocrine systems [40]
		Rb2	Diabetes, obesity, tumor, photoaging, virus infection and cardiovascular problems [41]
		Rc	Metabolic syndrome [42]
		Rd	Neuroprotection [43], cardiovascular, cerebrovascular and nervous systems [44]
		Rg3	Anti-angiogenic [45], anticancer, anti-inflammatory, antioxidant, immunomodulatory [46]
		Rg5	Anticancer [47], antidiabetic [48,49], anti-inflammatory [50], anti-osteoarthritic [51], sedative and hypnotic [52], and cardioprotective effects [53]
		Rh2	Neuroprotective effect [54], anticancer [55]
		Compound K	Rheumatoid arthritis [56]
	PPT	Re	Diabetes mellitus, nerve, inflammation, cardiovascular disease, and cancer [57]
		Rg1	Neurological diseases, like Alzheimer’s disease [58,59]
oleanolic type	Rh3		Neuroprotective effect [60], anti-tumor [61]

## Data Availability

No new data were created or analyzed in this study. Data sharing is not applicable to this article.

## Abbreviations

The following abbreviations are used in this manuscript:

AP-1	Activating Protein-1
MAPK	Mitogen-activated Protein Kinase
MHC-I	major histocompatibility complex class-I
MMPs	matrix metalloproteinases
NF-κB	Nuclear Factor-κB
PEG	Polyethylene Glycol
PI3K/Akt	phosphoinositide 3-kinase/protein kinase B
PMA	Phorbol 12-myristate 13-acetate
PPD	protopanaxadiol
PPT	protopanaxatriol
RhoA/ROCK1	Ras Homolog Gene Family Member A/Rho-associated Coiled Protein Kinase 1
ROS	reactive oxygen species
STAT3	Signal Transducer and Activator of Transcription 3
TGF-β/Smad	Transforming Growth Factor Beta/Small Mother Against Decapentaplegic
